# Use of Matrix-Assisted and Laser Desorption/Ionization Time-of-Flight Technology in the Identification of *Aeromonas* Strains Isolated from Retail Sushi and Sashimi

**DOI:** 10.3390/pathogens13060432

**Published:** 2024-05-21

**Authors:** Luca Nalbone, Salvatore Forgia, Federico Pirrone, Filippo Giarratana, Antonio Panebianco

**Affiliations:** 1 Department of Veterinary Science, University of Messina, Polo Universitario Dell’Annunziata, Viale Giovanni Palatucci SNC, 98168 Messina, Italy; frgsvt95p29g273n@studenti.unime.it (S.F.); federicopirrone@hotmail.it (F.P.); fgiarratana@unime.it (F.G.); apanebianco@unime.it (A.P.); 2 Riconnexia srls, Department of Veterinary Science, University of Messina, Polo Universitario Dell’Annunziata, Viale Giovanni Palatucci SNC, 98168 Messina, Italy

**Keywords:** food microbiology, bacteria identification, VITEK MS, mass spectroscopy, foodborne pathogens

## Abstract

The genus *Aeromonas* includes well-known pathogenic species for fishes and humans that are widely distributed in the aquatic environment and foods. Nowadays, one of the main issues related to wild *Aeromonas* isolates is their identification at the species level, which is challenging using classical microbiological and biomolecular methods. This study aims to test MALDI-TOF MS technology in the identification of *Aeromonas* strains isolated from n. 60 retail sushi and sashimi boxes using an implemented version of the SARAMIS software V4.12. A total of 43 certified *Aeromonas* strains were used to implement the SARAMIS database by importing the spectra obtained from their identification. The original SARAMIS version (V4.12) failed to recognize 62.79% of the certified strains, while the herein-implemented version (V4.12_plus_) allowed the identification of all the certified strains at least to the genus level with a match of no less than 85%. Regarding the sushi and sashimi samples, *Aeromonas* spp. was detected in n. 18 (30%) boxes. A total of 127 colonies were identified at the species level, with *A. salmonicida* detected as the most prevalent species, followed by *A. bestiarum* and *A. caviae*. Based on the results of the present study, we could speculate that MALDI-TOF technology could be a useful tool both for the food industry to monitor product contamination and for clinical purposes to make diagnoses effectively and quickly.

## 1. Introduction

The genus *Aeromonas* includes a group of 36 Gram-negative bacteria species widely distributed in the aquatic environment, particularly in rivers, lakes, and sewage [1]. Epidemiological evidence shows how water acts as a vehicle for *Aeromonas* spread and is responsible for the wide range of organisms and foods in which it can be isolated [2].

*Aeromonas* is, in fact, well known as a pathogen of several freshwater and marine animals, causing even severe diseases, such as the “red leg” (septicemic disease) caused by *A. hydrophila* in frogs or “furunculosis” caused by *A. salmonicida* in salmonids, responsible for major economic losses to the aquaculture sector [3]. 

Besides water, *Aeromonas* is also widely detected in other environmental districts, such as soil and vegetables, as well as in different kinds of foods intended for human consumption [4,5,6,7,8,9,10]. 

Due to its wide distribution, humans can be easily exposed to *Aeromonas* through various routes of infection, resulting in even severe cases of gastroenteritis, primary and secondary septicemia in immunocompromised people, and severe wound infections in healthy people [4,11,12,13]. Epidemiological evidence suggests that children are the most susceptible population in which co-infections by *Salmonella* and *Campylobacter* are frequently observed (polymicrobial infection) [14]. Out of 36 Aeromonas species, 19 are considered pathogenic to humans; however, most of the infections are attributed to only 4 species: *A. caviae*, *A. dhakensis*, *A. veronii* biovar *sobria,* and *A. hydrophila* [15,16].

The foodborne outbreaks related to this bacterium are mainly related to the consumption of contaminated water and food, such as freshwater fish and shellfish, meats, and fresh vegetables [6,7,8,9,10,11,12]. Among these, ready-to-eat food such as sushi and sashimi could represent a relevant source of infection considering the use of raw fish (in sushi and sashimi) and often also raw vegetables (in sushi) in their preparation. Sushi and sashimi are traditional Japanese dishes whose consumption has increased annually worldwide, posing concerns about the magnitude of the global exposure. Even though *Aeromonas* represents a relevant risk for these types of products, only a little evidence of its prevalence in sushi and sashimi is available, and further studies are certainly desirable [6]. 

Despite its wide environmental distribution, the consequent diffusion in the food, and the pathogenicity in humans, *Aeromonas* is still recognized as a potential or emerging foodborne pathogen since only a few cases of human infection are documented. Several authors have hypothesized that the low number of infection reports could be related to the lack of routine tests, the difficulty in strain identification, and the frequent occurrence of polymicrobial infection [14].

In this regard, rapid and efficient identification of the *Aeromonas* isolates at the species level would allow better risk management under different perspectives. The limited accuracy of the available methods in the identification of the *Aeromonas* strains is related to the high phenotypic and genotypic similarity that some species show between them [17]. Traditional techniques used for its identification are based both on the use of culture media that allow a morphological evaluation of the isolates as well as biochemical tests used for their physiological characterization. These last methods have the disadvantage of generally being time-consuming and not very accurate [18]. Over the years, various molecular methods have been developed, such as DNA–DNA hybridization and housekeeping genes; although these methods are known to be highly accurate in the identification of *Aeromonas*, they are rarely used in routine analysis due to the high cost, long time required for analysis, and the need for highly trained staff [1]. Furthermore, although 16S rRNA gene sequencing is considered one of the most popular tools for bacterial species identification, due to the high sequence conservation of the 16S rRNA gene (97.8–100%) in the *Aeromonas* genus and the existence of several copies of the gene with intragenomic heterogeneity in some strains, the usefulness of the 16S rRNA gene for taxonomic analysis at the species level is limited [19]. 

A valid alternative to the conventional identification methods just described is the mass spectrometry (MS) technique based on MALDI-TOF (matrix-assisted and laser desorption/ionization time-of-flight) technology [20]. MALDI-TOF MS started to be routinely utilized as a first-line identification method in microbiology laboratories in the last 12–15 years [21,22]. This technology offers many advantages over conventional microbiological and molecular techniques, which include reliability and rapidness, as it takes only a few minutes to identify microbes; simplicity; cost-effectiveness; and no highly trained staff are needed [23,24]. MALDI-TOF technology is based on the detection of the mass-to-charge ratio of specific targets, the ribosomal proteins of the bacteria, which provide a unique mass spectrum of the microorganism within a short time [23]. To compare the mass spectra of unknown bacteria with the reference mass spectra, there are several commercial databases available, such as SARAMIS software that was used in the present study. The identification accuracy via MALDI-TOF depends upon the database, which, in general, at the species level, is above 90% [25]. Therefore, an effective way to improve the accuracy of MALDI-TOF instrument identification is to expand and update the database [21]. As reported by several studies on the identification of bacteria of the *Aeromonas* genus, MALDI-TOF technology correctly identifies isolates at the genus level but shows variable reliability in the identification at the species level depending on the species examined [3,22,26]. 

Against this background, the present study aims to test MALDI-TOF MS technology in the identification of *Aeromonas* strains isolated from retail sushi and sashimi boxes using an implemented version of the SARAMIS software.

## 2. Materials and Methods

### 2.1. Experimental Plan

The experimental plan of the present study consisted of three different steps:

1. Evaluation of SARAMIS software–V4.12 in *Aeromonas* identification;

2. Implementation of SARAMIS Database for *Aeromonas* identification and evaluation of its efficiency;

3. Evaluation of *Aeromonas* spp. in retail sushi and sashimi boxes.

The steps are detailed below.

### 2.2. Preparation of the Bacterial Colonies for Step 1 and Step 2

Table 1 reports the n. 43 certified strains of different species belonging to the genus *Aeromonas* used in steps 1 and 2, as described below.

In detail, n. 29 (67.44%) certified strains were isolated from human clinical specimens, while n. 14 (32.56%) strains were isolated from environmental, food, or animal sources. The strains were kept frozen in Brain Heart Infusion Broth (Biolife, Milan, Italy) + 15% glycerol (Sigma-Aldrich, St. Louis, MO, USA) at − 80 °C at the microbial collection of the “Food Microbiology Laboratory” of the Department of Veterinary Sciences, University of Messina (Messina, Italy).

### 2.3. Evaluation of SARAMIS Software—V4.12 in Aeromonas Identification

The available version of the SARAMIS Knowledge Base software, V4.12, was used. n. 15 “Reference-Spectra” and n. 18 “Super-Spectra” of Aeromonas were present in the database (Table 2).

According to the SARAMIS manual [27], the following explanation of “Reference-Spectra” and “Super-Spectrum” are given: in Reference Spectra (or Consensus Spectra), only those mass signals with high frequency in a batch of spectra are recorded; thus, if the frequency threshold is set to 70%, only those mass signals recorded in at least 70% of the compared spectra are selected. The differences among spectra are due to the variability between isolates, the variability of mass spectra of single isolates, and analytical deviations. In Reference Spectra, these differences are eliminated, and only a reduced number of conserved mass signals is retained. The remaining masses are thus typically recorded in mass spectra of the particular taxon, irrespective of the isolates’ origin and cultivation conditions. In Reference Spectra, mass signals’ relative intensities are averaged. The Super Spectrum is created from a Reference Spectrum by assigning peak weights to each mass signal. Peak weights are generally higher for species-specific mass signals and lower for mass signals that are specific only at higher taxonomic levels, such as genera or families. Since the latter mass signals have no pertinence for species identification, the peak weights are set low, or the mass signals are even ignored by setting the peak weight to zero. Super Spectra are therefore highly specific artificial mass spectra that allow the unambiguous identification of an unknown isolate when its mass spectrum shows the specific mass pattern of a particular Super Spectrum.

To carry out a preliminary evaluation of the robustness and efficiency of SARAMIS software V4.12 in the identification of strains belonging to the *Aeromonas* genus, all the n. 43 certified strains reported in Table 1 were tested following the procedure reported below.

Once thawed, a total of 20 µL of each strain from the frozen stock was inoculated into 10 mL of Tryptone Soy Broth (TSB; Biolife, Milan, Italy) and incubated overnight at 30 °C. The broth cultures were then plated using a 10 µL loop onto Tryptone Soy Agar (TSA) (Biolife, Milan, Italy), TSA + 5% defibrinated mutton blood (TSAS; Biolife, Milan, Italy) (medium reported as optimal for identification), and incubated at 30 °C for 24 h to be tested with VITEK MS (bioMérieux Italia, Florence, Italy), automated device at our disposal for analyses based on MALDI-TOF MS technology.

At the same time, *Escherichia coli* ATCC 8739 (reference strain for calibration of the VITEK MS device) was prepared on TSAS and incubated at 37 °C for 24 h. The identification analyses were performed using disposable plates VITEK MS-DS with 48 spots (bioMérieux Italia, Florence, Italy) using the G3 and G4 as calibration spots for *E. coli* ATCC 8739.

Isolated colonies from each certified strain were picked using a sterile 1 µL loop from each medium plate, taking care not to unintentionally withdraw agar. Each picked colony was smeared in the center of a spot, and then 1 µL of alpha-cyano-4-hydroxy-cinnamic acid (CHCA) MALDI matrices (bioMérieux Italia, Florence, Italy) was added. The matrix/microorganism suspension was allowed to dry and crystallize completely and then placed in the acquisition station and loaded into the VITEK-MS. Crystallization was determined by visually checking the formation of crystals as a yellowish film on the spot surface. The VITEK-MS was used with the following settings: positive linear mode, laser frequency of 50 Hz, acceleration voltage of 20 kV, and extraction delay time of 200 ns. The mass spectra range was set to detect from 2000 to 20,000 Da. A unique mass spectrum was generated for each tested colony, which was transferred into SARAMIS software and compared to the database of Reference Spectra and Super Spectra. For each strain, the spectra of 3 colonies grown in each culture medium were acquired.

For the interpretation of the results returned by the SARAMIS software, confidence levels were established based on the match percentage between the spectra of the colonies tested and the spectra in the database: a match ≥99.9% was considered as an “excellent identification”, a match ranging between 60% and 99.8% was considered as a “good identification”, while a match <60% was interpreted as “no identification” [24].

### 2.4. Implementation of SARAMIS Database for Aeromonas Identification and Evaluation of Its Efficiency

For creation of new Super Spectra or implementation of existing ones, the overnight TSB culture of each of the n. 43 certified strain was plated using a 10 µL loop in four different growth media, including two selective media for growth of *Aeromonas* spp.: (i) Aeromonas starch DNA agar base (Biolife, Milan, Italy) (AEStarch); (ii) Pseudomonas Aeromonas Selective Agar Base acc. to KIELWEIN (GSP) (Merck, Darmstadt, Germany) supplemented with sodium penicillin G (10 mg/L; IGN Biomedicals, OH, USA); and two nonselective media: (iii) TSA; (iv) TSAS. All plates were incubated at 30 °C for 24 h. At the same time, the calibration strain *E. coli* ATCC 8739 was prepared on TSAS and incubated at 37 °C for 24 h.

For each *Aeromonas* certified strain, n. 4 colonies from each of the four media (a total of 16 colonies) were collected and processed for spectra acquisition, as described above (see Section 2.3) in order to increase the variability and the number of spectra to compare (16 for each strain) so that all possible mass variants resulting from both intra-species genetic differences and different culture conditions could be included in the spectra.

Through the data loading system “Target manager” in SARAMIS software, for each processed colony, the “genus”, “species”, and “type” corresponding to the identification number of the certified strain, “medium of origin”, and “incubation temperature” were reported. These inserted data automatically determined the creation within SARAMIS software of the respective “species” and “type” folders within the “Aeromonas” genus folder. The acquired spectra were subjected to an initial selection maintaining only those that fell within a range of 100 to 200 peaks, eliminating those with a number of peaks less than 100 and greater than 200 according to SARAMIS manual. Then, a dendrogram of the selected spectra was created using a specific tool in SARAMIS software. Only spectra with a similarity of 70% between duplicates and 65% between species were selected, while those with lower similarity were eliminated. Selected spectra were further compared to each other to determine the most frequently occurring mass signals. Only spectra with 60% of the masses in common were maintained, while all the other strains below the threshold were eliminated. The remaining spectra with a total of 40 mass signals in common represented the new Reference Spectra that were loaded into the SARAMIS database. To create Super Spectra from Reference Spectra, it was necessary to determine mass signals specific to the selected species by distinguishing conserved masses that were not at a higher taxonomic level (e.g., genus). In this regard, a comparison of the mass signals between the implemented Reference Spectra and the spectra of *Aeromonas* genus was performed, and the masses in common were excluded in order to create new Super Spectra of the considered species maintaining only characteristic mass signals. Finally, the specificity of the mass signals was evaluated by running a comparison of the newly created Super Spectra against the entire database since individual mass signals can also occur in spectra of different taxa. The new Super Spectra met two basic conditions: (i) the sum of peak weights was not more than 1400 points; (ii) the sum of peak weights of the mass signals in common with other taxa did not exceed 600 points.

The 43 certified strains already evaluated with the original version of SARAMIS software (see Section 2.3) were analyzed once again using the newly implemented version (V4.12_plus_).

### 2.5. Evaluation of Aeromonas spp. in Retail Sushi and Sashimi Boxes

#### 2.5.1. Sampling of Retail Sushi and Sashimi Boxes

A total of 60 retail sushi and sashimi boxes, including 5 different formulations, were purchased from different retailers in Sicily (southern Italy). Each box represented a sample and, in detail, the following were collected: 10 sushi-nigiri boxes (ingredients: raw salmon, rice, rice vinegar, and soy sauce), 10 sushi-hosomaki boxes (ingredients: nori seaweed, rice, rice vinegar, soy sauce, and salmon), 10 sushi-uramaki boxes (ingredients: sesame seeds, rice, rice vinegar, nori seaweed, soy sauce, salmon, and avocado), 15 salmon sashimi boxes (ingredients: raw salmon and soy sauce), and 15 tuna sashimi boxes (ingredients: raw tuna and soy sauce). The boxes contained 6 pieces each of the corresponding formulation.

Half of the nigiri, hosomaki, and uramaki boxes were prepared at the time of sale directly in-store, while the other half were sold already packaged and prepared at an industrial level.

At the time of sampling, boxes were stored in refrigeration regime and, once purchased, were transported inside coolers to the “Food Microbiology Laboratory” of the Department of Veterinary Sciences, University of Messina (Messina, Italy), and immediately analyzed as follows.

#### 2.5.2. Microbiological Analysis: Aeromonas Detection and Enumeration

The protocol adopted for the detection and enumeration of presumptive *Aeromonas* spp. was inspired by Lee et al. [7]. In detail, the sushi and sashimi pieces of each box were homogenized, and a representative sample of 10 g was aseptically put into a stomacher bag, diluted in a ratio of 1:9 *w*/*v* with sterile peptone water (Biolife, Milan, Italy), and homogenized through a stomacher (400 Circulator; International PBI s.p.a., Milano, Italy) for 60 s at 230 rpm. The homogenate was decimal diluted and plated onto GSP agar and incubated at 30 °C for 24–48 h. The typical yellow colonies of *Aeromonas* spp. were visually enumerated.

A maximum of 10 colonies per sample were collected using a sterile loop, streaked onto TSA, and incubated at 30 °C for 24–48 h. The isolations obtained were then identified by MALDI-TOF MS using the same settings reported in Section 2.4. through our implemented version of SARAMIS software (V4.12_plus_).

### 2.6. Data Analysis

The data acquired were presented as mean ± standard deviation or parts of the whole as percentages.

The normal distribution of the data of the bacterial loads detected in the sushi and sashimi samples was tested using the D’Agostino–Pearson omnibus test, and any significant differences between the different sushi samples were tested using ordinary one-way analysis of variance (ANOVA) or Welch’s *t*-test.

The critical level of significance (*p*) was set at 5% (0.05), and the test was performed two-tailed using Graph Pad Prism 9.1.1 software (Graph Pad Prism, San Diego, CA, USA).

Descriptive statistics was performed using Excel (V. 2022, Microsoft Corporation, Washington, WA, USA).

## 3. Results

### 3.1. Preliminary Assessment of SARAMIS V4.12 Performance in Aeromonas Identification

The results of the identifications obtained by SARAMIS software V 4.12 are shown in Table 3. Out of n. 43 strains tested, n. 27 (62.79%) were not even identified at the genus level, whereas the remaining n. 16 strains were identified as follows: n. 13 (30.23%) as *Aeromonas* sp., with an identification percentage ranging from 71.7% to 92.5%; n. 2 (4.65%) strains of *A. sobria* were identified, with an identification percentage ranging between 77.1% and 89.3%; while n. 1 strain of *A. veronii* (CECT4258) was misidentified both as *A. sobria* and *Aeromonas* sp.

### 3.2. Evaluation of the SARAMIS Version Implemented in Aeromonas Identification

The identification results of the *Aeromonas* strains processed using the implemented version of SARAMIS software (V4.12_plus_) proposed herein are shown in Table 4.

Version V4.12 was updated by adding new Super Spectra of species not previously present in the database (*A. allosaccharophila*, *A. enteropelogenes,* and *A. jandaei*) and implementing those of species already present (*A. bestiarum*, *A. caviae*, *A. hydrophila*, *A. salmonicida*, *A. sobria*, and *A. veronii*). Regarding the strains of *A. encheleia*, *A. media*, *A. molluscorum*, *A. popoffii*, *A. sanarellii*, *A. schubertii*, *A. taiwanensis,* and *A. tecta*, only Reference Spectra were added to those already existing, while no new Super Spectra were created, as only one strain of each was tested.

All tested strains were identified at least to the genus level (*Aeromonas* sp.) with an identification match of no less than 85% (good identification). The isolates of *A. encheleia*, *A. eucrenophila*, *A. media*, *A. molluscorum*, *A. popoffii*, *A. sanarellii*, *A. schubertii*, *A. taiwanensis,* and *A. tecta* were identified as *Aeromonas* sp., improving the previous identification with V4.12, according to which no match was observed. For all strains for which new Super Spectra were created and implemented, identification reached the species level for those species previously identified as *Aeromonas* sp. (*A. caviae*, *A. enteropelogenes*, *A. hydrophila*, and *A. veronii*) or not identified (*A. allosaccharophila*, *A. bestiarum*, *A. jandaei*, *A. salmonicida*, and *A. veronii*) with V4.12 and improved for those strains already identified at the species level (*A. sobria*).

### 3.3. Evaluation of Aeromonas spp. in Retail Sushi and Sashimi Boxes

Overall, presumptive *Aeromonas* spp. was detected in n. 18 (30%) samples, with an average load of 2.93 ± 0.89 Log CFU/g ranging between 1 and 3.99 Log CFU/g.

**Figure 1:**
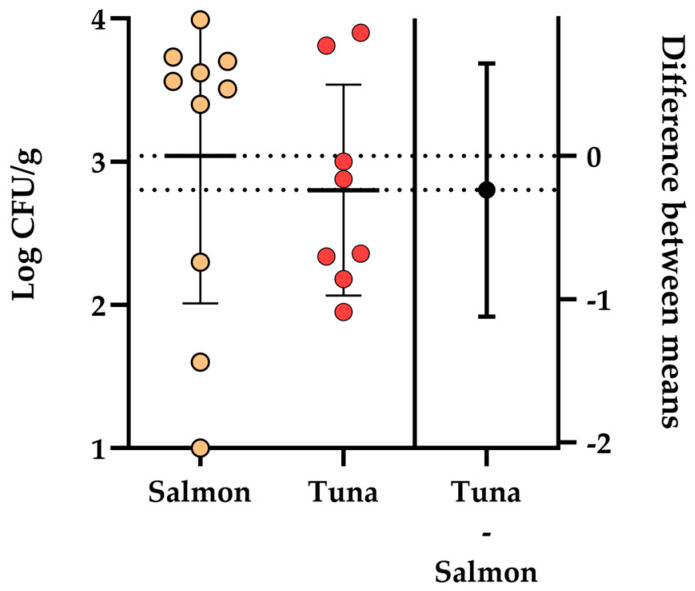
Estimation plot of the results obtained for the enumeration of presumptive *Aeromonas* spp. isolated from salmon and tuna sashimi sampled in different retailers in Messina (Southern Italy).

In detail, *Aeromonas* spp. was only detected in both salmon and tuna sashimi boxes, while it was not detected in any of the sushi samples (nigiri, hosomaki, and uramaki) (<10 CFU/g). Regarding the sashimi samples, *Aeromonas* spp. was detected in n. 10 (66.67%) salmon samples and 8 tuna samples (53.33%), with an average load of 3.04 ± 1.08 Log CFU/g and 2.80 ± 0.73 Log CFU/g, respectively. The loads ranged between 1 and 3.99 Log CFU/g in salmon sashimi samples and 1.95 and 3.90 Log CFU/g in tuna sashimi samples. The results of the statistical analysis revealed no significant difference in the average *Aeromonas* spp. load between salmon and tuna sashimi samples (*p* = 0.5753). The results obtained regarding the isolation and enumeration of *Aeromonas* spp. in sashimi samples are summarized in Figure 1.

A total of 164 colonies were tested by MALDI-TOF MS, and 148 colonies (90.24%) were identified as belonging to *Aeromonas* genus. In detail, 127 colonies (85.81%) were identified at the species level: the most prevalent was *A. salmonicida* (67.57%), followed by *A. bestiarum* (16.22%) and *A. caviae* (2.03%). The remaining 14.19% of the colonies were identified as *Aeromonas* spp.

The results arranged based on the type of sashimi samples (salmon or tuna) are shown in Table 5.

## 4. Discussions

Preliminary assessment of the SARAMIS V4.12 showed the poor efficiency of the software in the identification of different strains of the genus *Aeromonas,* possibly related to the few number of Reference Spectra and Super Spectra available in the database. Out of 43 strains tested, only the 2 strains (4.65%) of *A. sobria* were correctly identified at the species level (with a match ranging between 77.1% and 89.3%), n. 1 was misidentified as *A. sobria* instead of *A. veronii*, n. 13 (30.23%) were identified only at the genus level, and the remaining n. 27 (62.79%) were not identified.

In this regard, consulting the data in the literature, SARAMIS V4.12 showed less accuracy compared to other more updated MALDI-TOF MS databases. A comparison to identify clinical isolates of *Aeromonas* spp. documented by Kitagawa et al. between MALDI-Biotyper (Brucker Daltonics, Bremen, Germany) Flex Control software ver. 3.4 and VITEK MS and its analysis software VITEK MS ver. KB3.2 showed, for MALDI-Biotyper, correct identification at the genus level for all isolates. In contrast, the identification at the species level presented an important species-dependent variability, with an identification match of 0%, 0%, 72.3%, and 78.9% for *A. jandei*, *A. dhakensis*, *A. veronii,* and *A. caviae*, respectively, with the best result for *A. hydrophila* (93.3%). Regarding VITEK MS, similarly, all isolates were identified correctly at the genus level, but only *A. hydrophila* and *A. caviae* were accurately identified at the species level. Isolates of all other species (*A. jandei*, *A. dhakensis*, and *A. veronii*) were detected as more than one species [1].

Pérez-Sancho et al. reported that the software Biotyper Real-Time Classification v3.1 (Brucker Daltonics, Bremen, Germany) was able to identify all isolates of *Aeromonas* at the genus level and the species level, with identification match of 94.1%, 95.5%, 89.7%, and 96.3% for isolates of *A. bestiarum*, *A. hydrophila*, *A. salmonicida,* and *A. sobria*, respectively; whereas for *A. popoffi*, *A. media,* and *A. veronii,* the identification matches were 38.5%, 50%, and 71%, respectively. *A. dhakensis* and *A. piscicola* were misidentified as other species [3].

Porte et al. evaluated the use of two MALDI-TOF MS platforms (Microflex LT, from Bruker Daltonics; and Vitek MS, from BioMerieux) for the microbiological diagnosis of several microorganisms in a routine laboratory in Chile; the *Aeromonas* genus identification match at species level proved to be low, concluding that this technique has good precision in genus accuracy but with limited species differentiation [28].

In another clinical study, MALDI-TOF MS with BIOTYPER 2.0 (Bruker Daltonics, Bremen, Germany) correctly identified a high number of reference strains at the species level: *A. hydrophila* (35/35), *A. caviae* (19/23), *A. veronii* (6/6), and *A. aquariorum* (1/1) [29]. Teodoro et al. compared the identification of 60 isolates of *Aeromonas* spp. between PCR and MALDI-TOF MS with FlexControl software: the results matched 92.86% (13/14) for A. caviae, 84.62% (11/13) for *A. hydrophila*, 83.33% (5/6) for *A. veronii,* and 70.37% (19/27) of *Aeromonas* sp. [10].

Based on the identification results obtained using the implemented version of SARAMIS software (V4.12_plus_) proposed herein, we could speculate that, with an appropriate upgrade, MALDI-TOF technology based on VITEK MS and SARAMIS software can represent a rapid and relatively les-expensive method for the identification of bacterial colonies morphologically ascribable to *Aeromonas* spp. Although 16S rRNA gene sequencing and DNA-DNA hybridization, which is considered the gold standard method, allow for the identification of a greater number of species, the results of this study suggest that MALDI-TOF MS may be a valid, first-line identification system, reserving the use of molecular methods for those cases where the instrument fails to recognize isolates at the species level [30]. This would undoubtedly be a benefit in those routine situations where rapid identification is required. In this regard, there may be many fields of application for the instrument.

Contaminated foods represent one of the main sources of *Aeromonas* infection for humans, and several studies in the literature document an increasing occurrence of the bacterium in ready-to-eat foods, particularly in seafood, such as sushi and sashimi [4,6,7,8,10]. From this perspective, we investigated the occurrence of *Aeromonas* in retail sushi and sashimi boxes, detecting a prevalence of 30%. There is a wide variability in the prevalence of Aeromonas in retail sushi and sashimi reported in the literature.

The prevalence observed herein was relatively lower than that reported by Lee et al. in a similar study conducted on 30 retail sushi boxes purchased in Norwegian markets in which 18 *Aeromonas* strains, all identified as *A. media*, were isolated in 17% of the samples analyzed, with average loads of 1.5 Log CFU/g [7]. A higher prevalence was instead detected in another study conducted on 21 retail sushi products purchased in Italy, which isolated *Aeromonas* spp. in 90.5% of the samples [31]. Hoel et al. also reported a higher prevalence (71%) of *Aeromonas* spp. in nigiri and maki sushi compared to that observed herein, detecting an average load of 3 ± 0.3 Log CFU/g [32]. The similar load of 2.93 ± 0.89 Log CFU/g detected in the present study poses concerns considering the *A. hydrophila* concentration of 2.3–3 Log CFU/g estimated in a cold dish identified as the source of a Chinese outbreak affecting 349 people in 2012 [33]. However, using SARAMIS version V4.12_plus_, MALDI-TOF technology did not identify any *A. hydrophila* among the isolates, which instead were *A. salmonicida* (67.57%), *A. bestiarum* (16.22%), and *A. caviae* (2.03%). *A. salmonicida* is the only psychrophilic species among those isolated, and this could explain the higher prevalence since the retail sushi was kept at a refrigeration temperature at the time of sampling. Interestingly, there is evidence that 25 °C represents the temperature limit beyond which *A. salmonicida* cannot survive [14]. This contrasts with the results obtained in the present study, as the protocols adopted herein for the isolation of *Aeromonas* spp. involved an incubation temperature of the growth media of 30 °C. However, recent studies have proposed a new classification for *A. salmonicida* distinguishing between the subspecies that do not grow at temperatures above 37 °C (*A. salmonicida* subsp. *salmonicida*, *smithia*, *achromogenes* and *masoucida*) and the *A. salmonicda* subsp. *pectynolityca* that grows well even at 37 °C [14]. Against this background, we could speculate that *A. salmonicda* subsp. *pectynolityca* could be the one isolated in the present study.

The same species were identified through PCR analysis in retail sushi by Hoel et al., who isolated 118 *Aeromonas* strains using starch ampicillin agar incubated at 37 °C, identifying *A. salmonicida* as the most prevalent species (74%), followed by *A. bestiarum* (9%) and *A. caviae* (5%) [34]. Hoel et al. considered all isolates as potentially pathogenic due to the high prevalence of genes encoding different pathogenic factors (hemolysin, aerolysin, cytotoxic enterotoxin, and heat-labile and heat-stable cytotonic enterotoxin) posing concerns for the findings of the present study [34]. Among the isolated species, only *A. caviae* is part of a subset of species most implicated in human infections, together with *A. hydrophila*, *A. dhakensis,* and *Aeromonas veronii* biovar *sobria* [6]. The species *A. salmonicida* and *A. bestiarum* are basically considered primary pathogens of fishes, especially in salmonid culture systems and freshwater fishes, respectively [35,36]; however, several recent reports have highlighted the zoonotic potential of *A. salmonicida* [35].

Despite the significant evidence of the pathogenic effects on aquatic organisms and humans, the role of *Aeromonas* as a true foodborne pathogen continues to be questioned. Overall, the occurrence of *Aeromonas* in food has been mostly related to contamination with contaminated water [37]. The common physical–chemical treatments used for the purification of drinking water in a food plant are normally capable of devitalizing *Aeromonas*; however, ineffective water supply management could be responsible for its contamination. Furthermore, the preparation of sushi involves numerous manipulations and, therefore, the routes of exposure to contaminated water can be several and related to improper processing or hygiene practices. A further significant source of *Aeromonas* for sushi and sashimi is represented by fish, as intra-vitam contamination from the aquatic environment is certainly possible [38]. Interestingly, in the present study, *Aeromonas* was only detected in sashimi (raw fish) samples, while it was not detected in the more elaborate sushi, such as nigiri, hosomaki, and uramaki, in which even other ingredients (e.g., vegetables) could act as sources of contamination. This result could be related to the effect of the acidification of the rice that, in combination with low storage temperatures, supports *Aeromonas* growth [39]. It should also be considered that, although most of the *Aeromonas* species pathogenic for humans are mesophilic, such as *A. caviae* herein detected, they still manage to replicate at low temperatures, such as those at which retail sushi is kept on the market. Against this background, the choice of raw materials of high quality and the maintenance of the cold chain, as well as a proper application of good manufacturing and hygiene practices, are crucial for producing safe and quality sushi and sashimi.

As previously stated, there are only a few reports of human *Aeromonas* outbreaks associated with food consumption, and detailed information regarding infection doses is lacking [6]. Data collected during *Aeromonas* outbreaks that occurred in Norway and Sweden suggested an infection dose ranging between 10^6^ and 10^8^ cells, which is much higher than the average load (2.93 ± 0.89 Log) detected herein. However, other studies reported lower infective doses of 10^3^ to 10^4^ cells [40]. The current knowledge from clinical studies and recent outbreaks suggests that, depending on host–microbe interactions, even exposure to low and moderate doses of pathogenic *Aeromonas* can lead to infections, and the prevalence is probably underestimated due to many cases remaining undiagnosed [6]. Further concerns arise from the growing detection of multi-resistant *Aeromonas* strains [41] for which, in addition to finding effective contrast strategies, MALDI-TOF technology could be proposed as a valid and innovative support in the identification of resistant strains through a proteomic approach [42,43,44].

Against this background, the use of MALDI-TOF technology could be useful both for the food industry to monitor product contamination and for clinical purposes to make diagnoses effectively and quickly.

## 5. Conclusions

The results of the present study highlighted how MALDI-TOF technology can be a suitable tool for the rapid and effective identification of wild strains of *Aeromonas* isolated from food matrices. Furthermore, the detection of strains pathogenic to humans in sushi and sashimi samples available on the market stresses the need not to underestimate *Aeromonas* risk. Although the investigations were conducted only on *Aeromonas* strains, the results obtained and the simplicity of the analytical approach allow us to speculate on the use of MALDI-TOF for the routine identification of other pathogenic bacteria, not only in the food sector.

## Figures and Tables

**Table 1:** List of the certified *Aeromonas* strains used in the present study to implement SARAMIS software version V4.12.

Species	Sample Name	Source
*A. allosaccharophila*	CECT 4199^T^	Eel (*Anguilla anguilla*)
*A. allosaccharophila*	CECT 4220	Feces from patient with diarrhea
*A. allosaccharophila*	CECT 4911	Feces from patient with diarrhea
*A. allosaccharophila*	CECT 4912	Feces from patient with diarrhea
*A. bestiarum*	NCIMB 1134	Rainbow trout
*A. bestiarum*	DSM 13956^T^	Infected fish
*A. bestiarum*	CECT 5233	Feces from patient with diarrhea
*A. caviae*	NCIMB 882	Goldfish (*Crassius auratus*)
*A. caviae*	CECT 838^T^	Epizootic of young guinea pigs
*A. caviae*	CECT 5237	Feces from patient with diarrhea
*A. caviae*	CECT 5241	Feces from patient with diarrhea
*A. encheleia*	DSM 11577^T^	Healthy eel in fresh water
*A. enteropelogenes*	CECT 4255^T^	Human feces
*A. enteropelogenes*	CECT 4487^T^	Human feces
*A. enteropelogenes*	CECT 4936	Feces from patient with diarrhea
*A. enteropelogenes*	CECT 4937	Feces from patient with diarrhea
*A. eucrenophila*	DSM 17534^T^	Fresh water fish
*A. hydrophila*	ATCC 7966^T^	Milk
*A. hydrophila*	CECT 398	Human feces of a child with diarrhea
*A. hydrophila* sub. *dhakensis*	CECT 5743	Feces from patient with diarrhea
*A. hydrophila* sub. *dhakensis*	CECT 5744	Feces from patient with diarrhea
*A. hydrophila* sub. *dhakensis*	CECT 5745	Feces from patient with diarrhea
*A. jandaei*	CECT 4228^T^	Feces from patient with diarrhea
*A. jandaei*	CECT 4813	Feces from patient with diarrhea
*A. jandaei*	CECT 4815	Feces from patient with diarrhea
*A. media*	DSM 4881^T^	Fish farm effluent
*A. molluscorum*	CECT 5864	Wedge shells (*Donax trunculus*)
*A. popoffii*	DSM 19604^T^	Drinking water
*A. salmonicida*	NCIMB 1102^T^	Atlantic salmon
*A. sanarellii*	CECT 7402	Human wound
*A. schubertii*	CECT 4240^T^	Forehead abscess
*A. sobria*	NCIMB 75	Diseased freshwater fish
*A. sobria*	CECT 4245^T^	Fish
*A. taiwanensis*	CECT 7403	Human wound
*A. tecta*	CECT 7083	Feces from patient with diarrhea
*A. veronii*	CECT 4258	Feces from patient with diarrhea
*A. veronii*	CECT 4259	Feces from patient with diarrhea
*A. veronii*	CECT 4904	Feces from patient with diarrhea
*A. veronii*	CECT 4906	Feces from patient with diarrhea
*A. veronii*	CECT 4907	Feces from patient with diarrhea
*A. veronii*	CECT 4908	Feces from patient with diarrhea
*A. veronii*	CECT 4910	Feces from patient with diarrhea
*A. veronii* biovar *veronii*	CECT 4257^T^	Sputum of drowning victim

**Table 2:** Reference Spectra and Super Spectra list of *Aeromonas* species included in SARAMIS Database V4.12.

n.	Reference Spectra	n.	Super Spectra
1	*Aeromonas bestiarum*	1	*Aeromonas bestiarum*
2	*Aeromonas eucrenophila*	2	*Aeromonas encheleia*
3	*Aeromonas hydrophila*	3	*Aeromonas eucrenophila*
4	*Aeromonas hydrophila* ssp. *hydrophila*	4	*Aeromonas hydrophila*
5	*Aeromonas hydrophila/caviae*	5	*Aeromonas media*
6	*Aeromonas punctata* ssp. *caviae*	6	*Aeromonas molluscorum*
7	*Aeromonas punctata* ssp. *punctata*	7	*Aeromonas popoffi*
8	*Aeromonas salmonicida* ssp. *masoucida*	8	*Aeromonas punctata*
9	*Aeromonas salmonicida* ssp. *salmonicida*	9	*Aeromonas punctata* ssp. *caviae*
10	*Aeromonas sobria*	10	*Aeromonas punctata* ssp. *caviae/punctata*
11	*Aeromonas* spp.	11	*Aeromonas salmonicida*
12	*Aeromonas tecta*	12	*Aeromonas schubertii*
13	*Aeromonas veronii*	13	*Aeromonas sharmana*
14	*Aeromonas veronii* biovar *sobria*	14	*Aeromonas simiae*
15	*Aeromonas veronii* biovar *veronii*	15	*Aeromonas sobria*
		16	*Aeromonas tecta*
		17	*Aeromonas trota*
		18	*Aeromonas veronii*

**Table 3:** Identification results of different *Aeromonas* strains processed using MALDI-TOF technology with identification software SARAMIS, version 4.12.

Species	Identification					
	TSA			TSAS		
	C 1	C 2	C 3	C 1	C 2	C 3
*A. allosaccharophila* (CECT 4199^T^)	n.i.	n.i.	n.i.	n.i.	n.i.	n.i.
*A. allosaccharophila* (CECT 4220)	n.i.	n.i.	n.i.	n.i.	n.i.	n.i.
*A. allosaccharophila* (CECT 4911)	n.i.	n.i.	n.i.	n.i.	n.i.	n.i.
*A. allosaccharophila* (CECT 4912)	n.i.	n.i.	n.i.	n.i.	n.i.	n.i.
*A. bestiarum* (NCIMB 1134)	n.i.	n.i.	n.i.	n.i.	n.i.	n.i.
*A. bestiarum* (DSM 13956^T^)	n.i.	n.i.	n.i.	n.i.	n.i.	n.i.
*A. bestiarum* (CECT 5233)	n.i.	n.i.	n.i.	n.i.	n.i.	n.i.
*A. caviae* (NCIMB 882)	*Aeromonas* sp. **77.8%**	*Aeromonas* sp. **80.0%**	*Aeromonas* sp. **79.3%**	*Aeromonas* sp. **78.1%**	*Aeromonas* sp. **78.1%**	*Aeromonas* sp. **78.1%**
*A. caviae* (CECT 838^T^)	n.i.	n.i.	n.i.	n.i.	n.i.	n.i.
*A. caviae* (CECT 5237)	n.i.	n.i.	n.i.	n.i.	n.i.	n.i.
*A. caviae* (CECT 5241)	n.i.	n.i.	n.i.	n.i.	n.i.	n.i.
*A. encheleia* (DSM 11577^T^)	n.i.	n.i.	n.i.	n.i.	n.i.	n.i.
*A. enteropelogenes* (CECT 4255^T^)	n.i.	n.i.	n.i.	n.i.	n.i.	n.i.
*A. enteropelogenes* (CECT 4487^T^)	n.i.	*Aeromonas* sp. **79.1%**	*Aeromonas* sp. **78.9%**	n.i.	n.i.	n.i.
*A. enteropelogenes* (CECT 4936)	*Aeromonas* sp. **76.3%**	n.i.	n.i.	n.i.	n.i.	n.i.
*A. enteropelogenes* (CECT 4937)	n.i.	*Aeromonas* sp. **77.1%**	n.i.	n.i.	n.i.	n.i.
*A. eucrenophila* (DSM 17534^T^)	n.i.	n.i.	n.i.	n.i.	n.i.	n.i.
*A. hydrophila* (ATCC 7966^T^)	*Aeromonas* sp. **86.3%**	*Aeromonas* sp. **76.9%**	*Aeromonas* sp. **88.9%**	*Aeromonas* sp. **92.5%**	*Aeromonas* sp. **90.9%**	*Aeromonas* sp. **78.3%**
*A. hydrophila* (CECT 398)	*Aeromonas* sp. **85.4%**	*Aeromonas* sp. **71.7%**	*Aeromonas* sp. **84.4%**	*Aeromonas* sp. **82.5%**	*Aeromonas* sp. **81.0%**	n.i.
*A. hydrophila sub. dhakensis* (CECT 5743)	n.i.	*Aeromonas* sp. **86.2%**	n.i.	*Aeromonas* sp. **85.5%**	*Aeromonas* sp. **86.3%**	*Aeromonas* sp. **86.0%**
*A. hydrophila sub. dhakensis* (CECT 5744)	*Aeromonas* sp. **76.2%**	n.i.	*Aeromonas* sp. **85.4%**	n.i.	n.i.	n.i.
*A. hydrophila sub. dhakensis* (CECT 5745)	*Aeromonas* sp. **91.8%**	*Aeromonas* sp. **79.3%**	n.i.	n.i.	n.i.	n.i.
*A. jandaei* (CECT 4228^T^)	n.i.	n.i.	n.i.	n.i.	n.i.	n.i.
*A. jandaei* (CECT 4813)	n.i.	n.i.	n.i.	n.i.	n.i.	n.i.
*A. jandaei* (CECT 4815)	n.i.	n.i.	n.i.	n.i.	n.i.	n.i.
*A. media* (DSM 4881^T^)	n.i.	n.i.	n.i.	n.i.	n.i.	n.i.
*A. molluscorum* (CECT 5864)	n.i.	n.i.	n.i.	n.i.	n.i.	n.i.
*A. popoffii* (DSM 19604^T^)	n.i.	n.i.	n.i.	n.i.	n.i.	n.i.
*A. salmonicida* (NCIMB 1102^T^)	n.i.	*Aeromonas* sp. **78.3%**	*Aeromonas* sp. **79.3%**	n.i.	n.i.	n.i.
*A. sanarellii* (CECT 7402)	n.i.	n.i.	n.i.	n.i.	n.i.	n.i.
*A. schubertii* (CECT 4240^T^)	n.i.	n.i.	n.i.	n.i.	n.i.	n.i.
*A. sobria* (NCIMB 75)	n.i.	*A. sobria* **89.3%**	*A. sobria* **87.6%**	n.i.	*A. sobria* **77.1%**	
*A. sobria* (CECT 4245^T^)	*A. sobria* **77.5%**	*A. sobria* **85.6%**	n.i.	n.i.	*A. sobria* **86.9%**	*A. sobria* **87.3%**
*A. taiwanensis* (CECT 7403)	n.i.	n.i.	n.i.	n.i.	n.i.	n.i.
*A. tecta* (CECT 7083)	n.i.	n.i.	n.i.	n.i.	n.i.	n.i.
*A. veronii* (CECT 4258)	*Aeromonas* sp. **79.3%**	n.i.	*A. sobria* **75.6%**	n.i.	*A. sobria* **77.3%**	n.i.
*A. veronii* (CECT 4259)	n.i.	n.i.	n.i.	n.i.	n.i.	n.i.
*A. veronii* (CECT 4904)	n.i.	*Aeromonas* sp. **89.3%**	n.i.	n.i.	n.i.	*Aeromonas* sp. **71.9%**
*A. veronii* (CECT 4906)	n.i.	n.i.	n.i.	n.i.	n.i.	n.i.
*A. veronii* (CECT 4907)	n.i.	*Aeromonas* sp. **77.5%**	n.i.	n.i.	*Aeromonas* sp. **77.4%**	n.i.
*A. veronii* (CECT 4908)	*Aeromonas* sp. **78.2%**	*Aeromonas* sp. **88.3%**	n.i.	n.i.	n.i.	n.i.
*A. veronii* (CECT 4910)	n.i.	n.i.	n.i.	n.i.	n.i.	n.i.
*A. veronii bv veronii* (CECT 4257^T^)	n.i.	n.i.	n.i.	n.i.	n.i.	n.i.

n.i. = no identification; TSA = Tryptic Soy Agar; TSAS = TSA + 5% defibrinated mutton blood.

**Table 4:** Comparison between the identification of different *Aeromonas* strains using SARAMIS software V 4.12 and an implemented version of the software (V4.12_plus_) proposed in the present study. The best percentages of strain identifications performed with both versions of the software are reported regardless of the type of growth medium used.

STRAINS	SARAMIS V4.12	SARAMIS V4.12_plus_
*A. allosaccharophila* (CECT 4199^T^)	n.i.	*A. allosaccharophila* **85.6%**
*A. allosaccharophila* (CECT4220)	n.i.	*A. allosaccharophila* **86.8%**
*A. allosaccharophila* (CECT4911)	n.i.	*A. allosaccharophila* **87.7%**
*A. allosaccharophila* (CECT4912)	n.i.	*A. allosaccharophila* **85.1%**
*A. bestiarum* (NCIMB 1134)	n.i.	*A. bestiarum* **89.7%**
*A. bestiarum* (DSM 13956^T^)	n.i.	*A. bestiarum* **85.4%**
*A. bestiarum* (CECT5233)	n.i.	*A. bestiarum* **86.1%**
*A. caviae* (NCIMB 882)	*Aeromonas* sp. **80.0%**	*A. caviae* **94.5%**
*A. caviae* (CECT 838^T^)	n.i.	A. caviae **87.9%**
*A. caviae* (CECT5237)	n.i.	*A. caviae* **87.0%**
*A. caviae* (CECT5241)	n.i.	*A. caviae* **86.3%**
*A. encheleia* (DSM 11577^T^)	n.i.	*Aeromonas* sp. **85.8%**
*A. enteropelogenes* (CECT 4255^T^)	n.i.	*A. enteropelogenes* **86.0%**
*A. enteropelogenes* (CECT 4487^T^)	*Aeromonas* sp. **79.1%**	*A. enteropelogenes* **89.8%**
*A. enteropelogenes* (CECT4936)	*Aeromonas* sp. **76.3%**	*A. enteropelogenes* **92.0%**
*A. enteropelogenes* (CECT4937)	*Aeromonas* sp. **77.1%**	*A. enteropelogenes* **93.4%**
*A. eucrenophila* (DSM 17534^T^)	n.i.	*Aeromonas* sp. **86.6%**
*A. hydrophila* (ATCC 7966^T^)	*Aeromonas* sp. **92.5%**	*A. hydrophila* **97.1%**
*A. hydrophila* (CECT 398)	*Aeromonas* sp. **85.4%**	*A. hydrophila* **94.7%**
*A. hydrophila sub. dhakensis* (CECT5743)	*Aeromonas* sp. **86.3%**	*A. hydrophila* **93.0%**
*A. hydrophila sub. dhakensis* (CECT5744)	*Aeromonas* sp. **85.4%**	*A. hydrophila* **92.6%**
*A. hydrophila sub. dhakensis* (CECT5745)	*Aeromonas* sp. **91.8%**	*A. hydrophila* **96.3%**
*A. jandaei* (CECT 4228^T^)	n.i.	*A. jandaei* **89.0%**
*A. jandaei* (CECT4813)	n.i.	*A. jandaei* **87.7%**
*A. jandaei* (CECT4815)	n.i.	*A. jandaei* **86.9%**
*A. media* (DSM 4881^T^)	n.i.	*Aeromonas* sp. **88.1%**
*A. molluscorum* (CECT5864)	n.i.	*Aeromonas* sp. **89.4%**
*A. popoffii* (DSM 19604^T^)	n.i.	*Aeromonas* sp. **91.0%**
*A. salmonicida* (NCIMB 1102^T^)	*Aeromonas* sp. **79.3%**	*A. salmonicida* **94.9%**
*A. sanarellii* (CECT7402)	n.i.	*Aeromonas* sp. **85.2%**
*A. schubertii* (CECT 4240^T^)	n.i.	*Aeromonas* sp. **85.7%**
*A. sobria* (NCIMB 75)	*A. sobria* **89.3%**	*A. sobria* **99.8%**
*A. sobria* (CECT 4245^T^)	*A. sobria* **87.3%**	*A. sobria* **99.0%**
*A. taiwanensis* (CECT7403)	n.i.	*Aeromonas* sp. **88.8%**
*A. tecta* (CECT7083)	n.i.	*Aeromonas* sp. **85.7%**
*A. veronii* (CECT4258)	*A. sobria* **79.3%**	*A. veronii* **86.1%**
*A. veronii* (CECT4259)	n.i.	*A. veronii* **89.2%**
*A. veronii* (CECT4904)	*Aeromonas* sp. **89.3%**	*A. veronii* **95.5%**
*A. veronii* (CECT4906)	n.i.	*A. veronii* **87.3%**
*A. veronii* (CECT4907)	*Aeromonas* sp. **77.5%**	*A. veronii* **93.4%**
*A. veronii* (CECT4908)	*Aeromonas sp*. **88.3%**	*A. veronii* **96.6%**
*A. veronii* (CECT4910)	n.i.	*A. veronii* **85.9%**
*A. veronii bv veronii* (CECT 4257^T^)	n.i.	*A. veronii* **88.9%**

n.i. = no identification.

**Table 5:** MALDI-TOF MS identifications of 148 presumptive Aeromonas colonies isolated from salmon and tuna sashimi sampled in different supermarkets and restaurants in Messina (Southern Italy).

Identification	Salmon Sashimi	Tuna Sashimi	Total
*Aeromonas* spp.	11 (13.92%)	10 (14.49%)	21 (14.19%)
*A. bestiarum*	6 (7.59%)	18 (26.09%)	24 (16.22%)
*A. caviae*	3 (3.80%)	0	3 (2.03%)
*A. salmonicida*	59 (74.68%)	41 (59.42%)	100 (67.57%)

## Data Availability

All the research data have been shared.
